# Numerical Evaluation of Abdominal Aortic Aneurysms Utilizing Finite Element Method

**DOI:** 10.3390/diagnostics15060697

**Published:** 2025-03-12

**Authors:** Konstantinos Kyparissis, Nikolaos Kladovasilakis, Maria-Styliani Daraki, Anastasios Raptis, Polyzois Tsantrizos, Konstantinos Moulakakis, John Kakisis, Christos Manopoulos, Georgios E. Stavroulakis

**Affiliations:** 1 School of Production Engineering and Management, Technical University of Crete, 731 00 Chania, Greece; kostaskuparissis@gmail.com (K.K.); nkladovasilakis@tuc.gr (N.K.); mdaraki1@tuc.gr (M.-S.D.); 2 Laboratory of Biofluid Mechanics & Biomedical Technology, School of Mechanical Engineering, National Technical University of Athens, 157 72 Zografos, Greece; raptistasos@mail.ntua.gr (A.R.); manopoul@central.ntua.gr (C.M.); 3 Faculty of Medicine, School of Health Sciences, University General Hospital of Patras ‘Agios Andreas’, 263 32 Patra, Greece; polyzois.tsantrizos@hotmail.com; 4 Department of Vascular Surgery, Attikon University Hospital, National and Kapodistrian University of Athens, 106 79 Athens, Greece; kmoulakakis@med.uoa.gr (K.M.); kakisis@med.uoa.gr (J.K.)

**Keywords:** abdominal aneurysms, aortic walls, 3D reconstruction, DICOM, finite elements models, mechanical behavior, rupture risk assessment

## Abstract

**Background:** In recent years, more and more numerical tools have been utilized in medicine in or-der to assist the evaluation and decision-making processes for complex clinical cases. Towards this direction, Finite Element Models (FEMs) have emerged as a pivotal tool in medical research, particularly in simulating and understanding the complex fluid and structural behaviors of the circulatory system. Furthermore, this tool can be used for the calculation of certain risks regarding the function of the blood vessels. **Methods:** The current study developed a computational tool utilizing the finite element method in order to numerically evaluate stresses in aortas with abdominal aneurysms and provide the necessary data for the creation of a patient-specific digital twin of an aorta. More specifically, 12 different cases of aortas with abdominal aneurysms were examined and evaluated. **Results:** The first step was the 3D reconstruction of the aortas trans-forming the DICOM file into 3D surface models. Then, a finite element material model was developed simulating accurately the mechanical behavior of aortic walls. **Conclusions:** Through the results of these finite element analyses the values of tension, strain, and displacement were quantified and a rapid risk assessment was provided revealing that larger aneurysmatic regions elevate the risk of aortic rupture with some cases reaching an above 90% risk.

## 1. Introduction

The human aorta is defined as the main blood vessel that starts from the upper part of the heart and is responsible for transporting blood pumped from the left ventricle into the rest of the circulatory system [1]. Since aortic diseases are often life-threatening, understanding aortic pathophysiology through research is crucial. Critical objects of the field are the compositional structure of the aortic wall and hemodynamics, such as blood flow type, flow velocity, blood pressure, and pulse rate [2]. Aneurysms are one of the primary pathologies impacting the aorta. Based on existing literature [3], aortic aneurysms are defined as the swelling and expansion of the surface (wall) of the blood vessel, specifically greater than 50% of the normal values of its diameter. This dilation is progressively manifested in parts of the aorta with a high risk of rupture, causing internal bleeding and death [4]. The aorta is divided into two sections: the thoracic aorta and the abdominal aorta. However, the abdominal aorta has a higher risk of aneurysm formation [5]. Therefore, by understanding the pathophysiology of the aorta, patient-specific clinical cases of abdominal aortic aneurysms can be modeled through specific parameterization.

**Figure 1:**
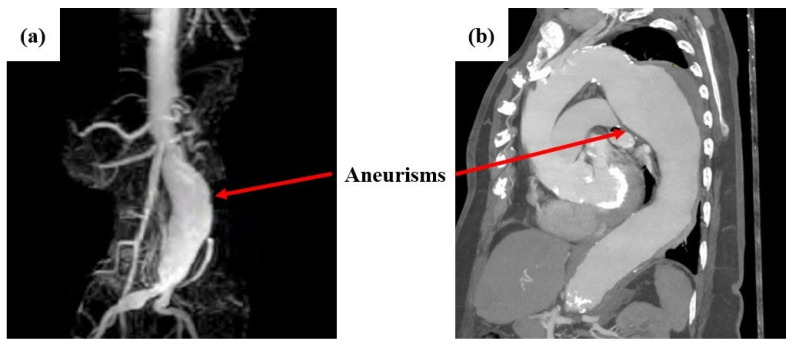
Indicative images of abdominal aortic aneurysms captured with: (**a**) MRA and (**b**) MRI imaging [9,10].

The causes of aneurysms are not predetermined, but factors such as heredity, atherosclerosis, diabetes, hypertension, or injuries from previous vascular surgery may contribute to their occurrence [6]. Usually, patients do not show symptoms as aneurysms are often incidental findings during tests. However, some of the most common symptoms include abdominal, chest, back, or groin pain, as well as palpitations in the abdomen in synchrony with the heartbeat. In rare cases, areas of blue color and pain may appear in the feet and toes due to the dispersal of debris from the aneurysm downward [7]. Aneurysms are usually diagnosed as incidental findings because patients are asymptomatic. They are typically detected through, computed tomography (CT) or angiography (CTA), triplex ultrasound, magnetic resonance imaging (MRI), or angiography (MRA). In very rare cases, when imaging tests are negative, but an aneurysm is strongly suspected, more specialized tests may be necessary (Positron Emission Tomography (PET) Scan, etc.) [8]. Indicatively, Figure 1 shows cases of abdominal aortic aneurysms captured with MRA (left) and MRI (right) imaging [9,10].

Over the last decade, simulation studies have been performed on aneurysmal aortic regions focusing both on hemodynamics and structural integrity of the examined aortas. In detail, the vast majority of published studies have investigated the flow stream inside the aorta and how it is influenced by wall abnormalities [11,12,13,14]. Thus, this aspect of computational analysis for aortic arteries has reached a sufficient technology readiness level. Therefore, nowadays, more and more studies are focusing on the structural integrity simulation of aortic wall behavior and assessment of the risk of rupture. This problem has increased complexity due to the exotic nature of the aortic tissue, which possesses dynamic characteristics depending on the patient’s age, gender, weight, etc. [15,16,17]. Towards this direction, fluid–structure interaction within realistic three-dimensional models of the aneurysmatic aortic section has been employed as guidance to assess the risk of rupture of the aneurysm [18,19]. In addition, Humphrey and Holzapfel [20] have summarized in a comprehensive review the mechanics, mechanobiology, and modeling of human abdominal aortic arteries and aneurysms, showing the need for patient-specific computational models. All these studies are capable of evaluating the mechanical behavior of the aortic wall and assessing the risk of potential rupture. However, the complexity of the aforementioned studies increased the execution time and the required computational power for each analysis. Therefore, there is a need to develop patient-specific computational tools utilizing finite element models that can produce reliable and sufficiently accurate results assisting the in-situ medical decision-making for the examined cases.

**Figure 2:**
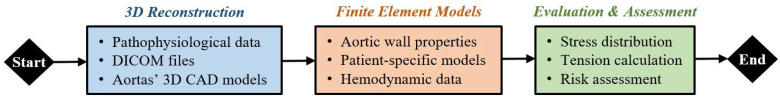
Flowchart of the current study.

In this context, the present study performed a numerical evaluation of aortas with abdominal aneurysm employing FEMs in order to rapidly extract reliable and accurate results assessing the risk of rupture. More specifically, geometrical data were acquired from CT scans of 12 abdominal aortas previously diagnosed with aneurysms and the analysis of the mechanical response under specific assumptions was performed using computational mechanics. The objective of this work is to develop and study aneurysmal aortas in a simulation environment using finite elements models employing static linear analysis of the aortic wall and then rapidly assess the rupture risk. The novelty of this paper is the provision of how the aneurysmal abdominal aortic region behaves under constant pressure to find and highlight the areas of maximum stresses, deformations, and displacements on the surfaces. Then, these acquired data were used in order to rapidly assess the risk of potential rupture, providing healthcare professionals with a reliable data-driven decision-making tool. It must be noted that predictions of stresses and strains in parts of the aortic artery that are not accessible can be provided, like a virtual sensor. The developed finite models were built and evaluated utilizing existing geometric data from the CT scans supplemented with assumptions on the values of the unknowns (material properties, arterial thickness, and arterial pressure) to demonstrate the capabilities of the mechanical computational models created. The results can be extended in several directions. This will pave the way for further study both in a theoretical setting and in clinical application. Figure 2 shows the flowchart of the current study.

## 2. Materials and Methods

### 2.1. Patient Data and 3D Reconstruction

In this study, retrospective anonymized CT imaging data in DICOM format from 12 cases with abdominal aortic aneurysms was provided from the Department of Vascular Surgery, Attikon University Hospital, Athens, Greece. Regarding the personal information, only the gender and the age of each patient were retrieved as these two parameters possess a significant role in the pathophysiology of the aorta, especially for the size of the aneurysm and the elasticity of the aortic walls. Table 1 lists these metrics for all selected cases along with a standard code name for each clinical case.

The next step was the 3D reconstruction of the patient aortas’ 3D models exploiting the acquired DICOM files. The steps have been presented in detail in reference [21], from which selected information is reproduced here. The first step in the segmentation and reconstruction procedure was the inspection of the CT scans and the selection of the most suitable imaging series to be further processed using RadiAnt DICOM Viewer (https://www.radiantviewer.com, accessed on 9 March 2025). The selected image s eries for each case consists of 2D medical images in DICOM format, which were imported into Mimics software (Materialise, Leuven, Belgium). By using brightness and contrast threshold tuning and colors, the required information has been extracted. Mimics software provides tools to perform an initial rough segmentation of the region of interest. First, a primary raw mask is created, which is processed further to provide an improved mask that serves as the final raw representation and a preliminary 3D model. Further processing of the model leads to a more accurate and smoother 3D representation, which is used for the creation of a 3D mesh with fewer imperfections. The outlined procedure, which is described in detail in [21], is necessary for the creation of a CAD model that can be used further for finite element analysis. Unfortunately, for the time being, this procedure cannot be fully automatized, a fact that makes the integration into an automatic Digital Twin environment difficult, i.e., a computer-generated replica of a patient’s aorta with an aneurysm capable to simulate and analyze the aorta’s behavior, assisting in assessing risks, plan treatments, and predict outcomes.

### 2.2. Finite Element Model

In order to simulate the mechanical behavior of the aortic walls with the abdominal aneurysms, the static module of the COMSOL Ver. 6 simulation platform was employed. More specifically, the isotropic elasticity model was utilized to capture the elastic mechanical behavior of the examined aortic regions. Isotropic elasticity material models provide sufficient accuracy for simulating the aortic wall, particularly up to a certain strain threshold before the exponential increase in deformation under higher loads. Notably, the exponential phase signifies the onset of aortic wall rupture. Moreover, linear elasticity models are more time-efficient and require lower computational power, aligning with the concept of the provision of rapid, data-driven decision-making tools. Through this process, the stress concentration regions were extracted along with the corresponding displacement, which indicates the potential risk for aortic rupture. The aortic wall material was simulated according to the mechanical properties that are listed in Table 2. These parameters were acquired from the existing literature [16,17,18]. Moreover, a mesh sensitivity analysis was performed focusing on equivalent von Mises stresses in order to obtain mesh-independent results. Regarding the computational mesh, tetrahedral elements were used with a minimum element size of 1 mm resulting number of elements between 150,000 and 200,000, depending on the examined aorta’s morphology. As boundary conditions, fixed surfaces were assumed in the inlet and outlet of the aortas’ model. On the other hand, as a loading condition universal static pressures were employed to simulate the difference between the low systolic and diastolic blood pressure, i.e., 130 mmHg and 80 mmHg, respectively, and the high systolic and diastolic pressure, i.e., 230 mmHg and 123 mmHg, respectively. The solver of the system executed the simulation in 10 incremental steps in order to ensure accurate and stable convergence of the solution. In the first step, the lowest difference of pressure was applied, i.e., ΔP = 130 − 80 = 50 mmHg, and the highest difference of pressure, i.e., ΔP = 230 − 123 = 77 mmHg, was applied in the last step achieving the maximum stresses, after their proportional increase by each step. Utilizing the approach with the pressure difference and knowing that in normal conditions aortic walls experience elastic deformations, the assumption of negligible geometric deformation of the aortic structure compared to the zero-pressure geometry is considered valid for this analysis [11].

The final step of the conducted numerical analysis was the assessment of rupture risk for the examined aortas. According to the existing literature [22,23], the rupture risk is defined as the probability of rupture of aortic walls, and it is highly correlated with the pressure–strain modulus. Moreover, the correlation between pressure–strain modulus and rupture risk probability follows a hyperbolic model, which is asymptotic to 1 (100%) [23], and an increase in pressure–strain modulus leads to an increase in the risk with an intense trend in the low values and smoother trend in the higher values. In the equation below, P_psm_ is the pressure–strain modulus, D_dias_ and D_sys_ are the diastolic and systolic critical diameter, and P_dias_ and P_sys_ are the diastolic and systolic pressure, respectively.

(1) $$P_{psm}=\frac{D_{dias}(P_{sys}-P_{dias})}{D_{sys}-D_{dias}}$$

## 3. Results

### 3.1. Extraction of the Aortas 3D Models

**Figure 3:**
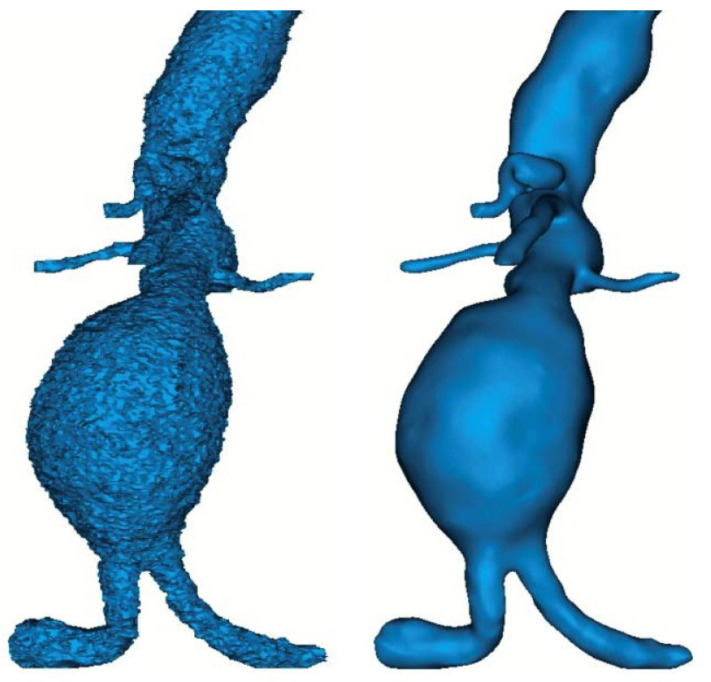
Indicative extracted 3D models before (**left**) and after (**right**) the noise removal.

Employing the aforementioned methodology of Section 2.1, surface 3D models were extracted for each examined clinical case. The 3D reconstructed aorta models were in the form of .STL format with fine triangle mashing in order to capture accurately the complex morphology of each aorta. Figure 3 illustrates indicative images of the reconstructed aortic geometry along with the smoothing process, which is necessary to the procedure due to the fact that CT scans extract the blood volume capturing at the same time high fluid noise that needs to be removed.

### 3.2. Finite Element Analysis Results

In this section, the main results of the static FEAs are presented for all 12 examined clinical cases. Several variations in aortic parameterization were tested in all geometries, and it was observed that the areas with the largest strains and deformations did not change. There may be fluctuations in the values at those points and in some cases, potentially adjacent areas on the surfaces may be affected. In conducting the research, it was observed that adequate modeling is one of the most crucial factors in performing simulations as the introduction of the 3D geometries and the parameterization defined directly affects the results. Several tests were needed to be able to simulate reality as best as possible as the geometry of the aorta is inherently complex. In addition, it is important to highlight that the physical characteristics of patients possess a crucial role in both modeling and inference. Furthermore, these observations significantly enhance our understanding of the underlying dynamics of aneurysmal aortic regions. The results presented do not show a direct correlation between arterial diameter and maximum stresses and presumably, there is a need to know more elements that characterize each case (for example, the length of the aneurysm or other morphological features).

**Figure 4:**
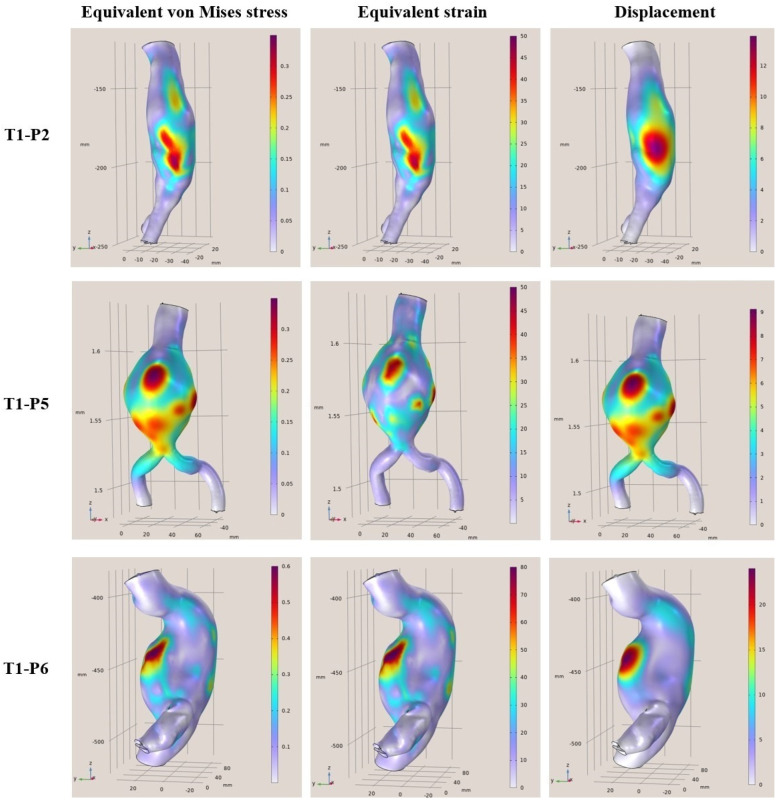
FEA results regarding the von Mises stress (MPa), strain and displacement (mm) distributions for T1-P2 (**Top**), T1-P5 (**Middle**), and T1-P6 (**Bottom**).

**Figure 5:**
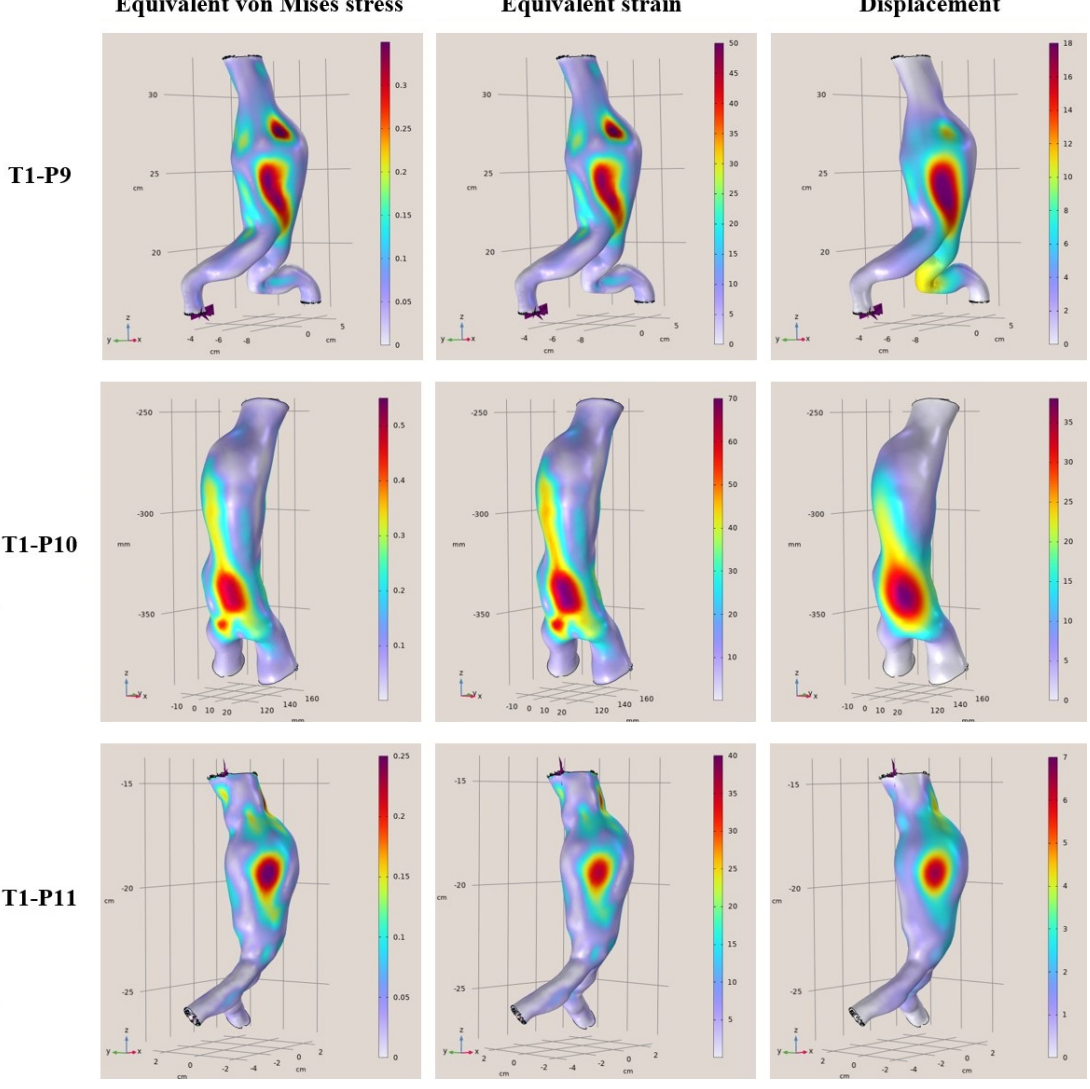
FEA results regarding the von Mises stress (MPa), strain, and displacement (mm) distributions for T1-P9 (**Top**), T1-P10 (**Middle**), and T1-P11 (**Bottom**).

**Figure 6:**
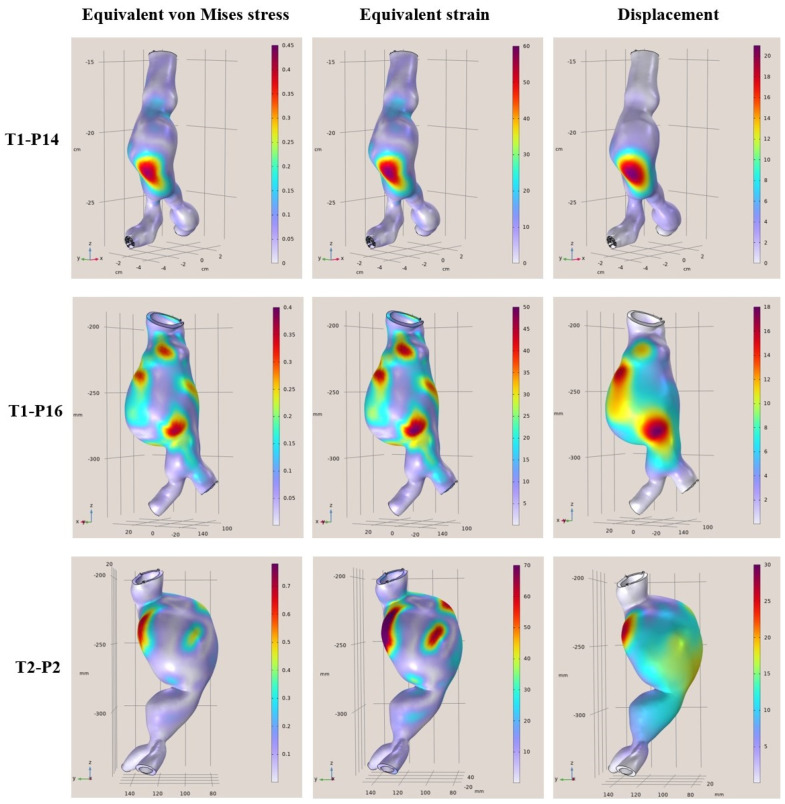
FEA results regarding the von Mises stress (MPa), strain, and displacement (mm) distributions for T1-P14 (**Top**), T1-P16 (**Middle**), and T2-P2 (**Bottom**).

**Figure 7:**
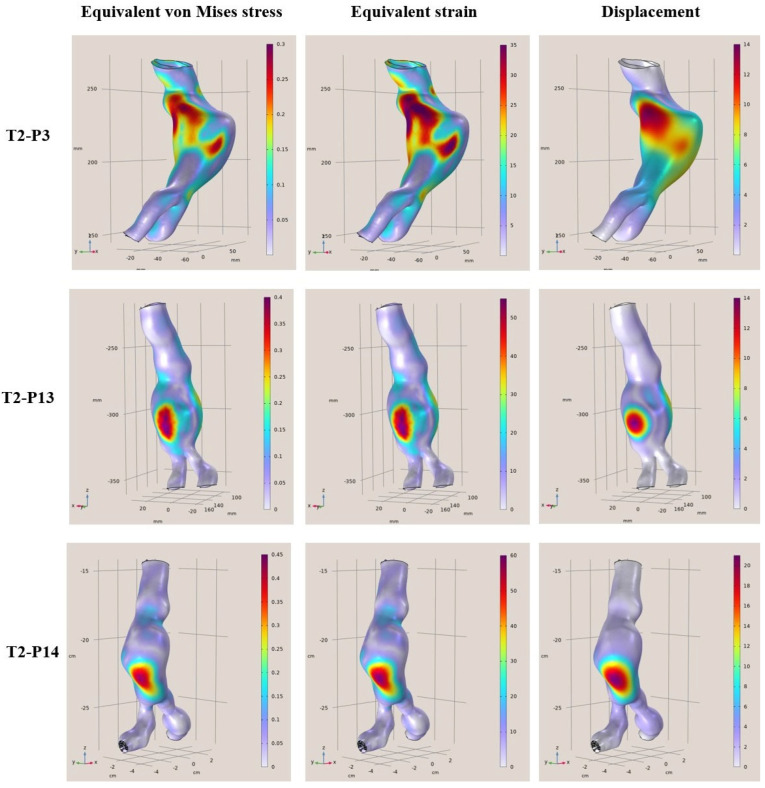
FEA results regarding the von Mises stress (MPa), strain, and displacement (mm) distributions for T2-P3 (**Top**), T2-P13 (**Middle**), and T2-P14 (**Bottom**).

More specifically, in Figure 4, Figure 5, Figure 6 and Figure 7, the von Mises stress (MPa), strain, and displacement (mm) distributions are illustrated for all 12 examined clinical cases. Furthermore, in Table 3, Table 4, Table 5 and Table 6, the main characteristics of examined aortic regions are listed, derived from the results of the analyses. It is observed that the largest values of von Mises stress, which are calculated from the components of the stresses acting on the shell surface, appearing on the surfaces of the models, are stresses arising at the points where the largest displacements and deformations occur on the shell surface.

Table 7 lists the observed systolic diameter for the maximum pressure, along with the pressure–strain modulus and the risk probability for each clinical case. Generally, as was expected, all samples revealed high rupture risk, i.e., above 50% due to the existence of aneurysmatic regions. However, the samples that experience large aneurysmatic regions, namely T1-P5, T1-P16, T2-P2, and T2-P3, possess a very high risk for wall rupture around 90% showing the larger the aneurysm the higher the rupture risk. Therefore, through comprehensive and fast FEAs is possible to extract efficient and reliable indicators regarding the probability of rupture aortic failure.

**Figure 8:**
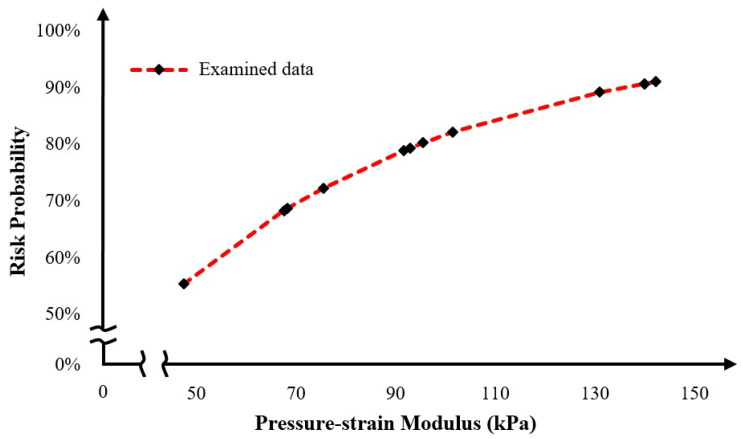
Graphical correlation between pressure–strain modulus and risk probability for the examined aortas.

In Figure 8, a graphical and virtual correlation between the pressure–strain modulus and rupture risk probability is presented. This diagram clearly shows a hyperbolic relationship between the two properties. Notably, following the trend of the curve, a pressure–strain modulus above 200 kPa is associated with a severe rupture risk probability exceeding 98%, indicating a high likelihood of aortic rupture. Conversely, values below the 50 kPa threshold appear to be more resilient to rupture scenarios.

## 4. Conclusions

To conclude, clinical cases from patients that are middle-aged to elderly from 57 to 81 years of age with a diagnostic diameter between 4.8 cm and 6.8 cm, were examined. Based on certain simplified assumptions like linearly elastic behavior, known support boundary conditions, and uniform blood pressure, it was derived that the areas where the greatest strains and displacements occur are considered to be areas of risk. In addition, the maximum stresses and strains differed from patient to patient, and it was concluded that each patient is different, the modeling was unique, and the maximum values changed for each patient case. Furthermore, based on the presented results, the maximum deviations ranged from 35% to 80% in some models. This means that the points where the maximum values occur on the shell surfaces are at a higher risk of aortic rupture under the pulsatile function. In terms of displacements, the maximum displacements from the surfaces appeared to range from 9 mm to 40 mm with the highest displacements occurring in the range 18 mm to 40 mm. Finally, regarding the rupture risk, larger aneurysmatic regions significantly increase the risk of aortic rupture, with some samples reaching a 90% risk, highlighting the effectiveness of comprehensive and fast FEAs in providing reliable rupture probability indicators. Through this process, the current paper provides a novel computational tool for rupture risk assessment that can assist in a more efficient and reliable decision-making process.

The correlation of aneurysm shape parameters with the arising stresses and strains—and the subsequent prediction of rupture risk—represents a promising proof-of-concept based on existing patient case studies. However, this initial study is subject to several limitations. Notably, the linear static analysis applied to a simplified shell structure does not fully capture the complex biomechanical behavior of the aortic wall. In particular, the model lacks the incorporation of non-linear material properties, the pulsatile dynamics of blood flow, and the detailed geometric features associated with thrombus formation and calcifications. These omissions/simplifications can significantly affect the accuracy of stress and strain estimations. A more robust approach would involve developing a parameterized aortic model—potentially leveraging statistical shape models—to automate the generation of diverse and realistic aortic geometries [24,25]. Such models should integrate advanced non-linear simulation techniques, dynamic pulsatile loading conditions, and enhanced anatomical detail to better mimic the anisotropic and layered structure of the aortic wall [26]. Addressing these aspects will be crucial for refining finite element analyses (FEAs) and advancing toward a fully automated, digital twin framework for personalized aortic rupture risk assessment.

## Figures and Tables

**Table 1:** Code name, gender, and age of each abdominal aortic aneurysm case.

Clinical Cases	Gender	Age	Clinical Cases	Gender	Age
**T1-P2**	M	81	**T1-P14**	M	81
**T1-P5**	M	72	**T1-P16**	M	76
**T1-P6**	M	66	**T2-P3**	M	78
**T1-P9**	M	57	**T2-P2**	M	66
**T1-P10**	M	57	**T2-P13**	M	62
**T1-P11**	M	70	**T2-P14**	M	67

**Table 2:** Parameters of isotropic linear elastic material.

Properties	Values
Density	1095 kg/m^3^
Young’s modulus	0.7 MPa
Poisson ratio	0.45

**Table 3:** Characteristics of examined aortas: T1-P2, T1-P5, and T1-P6.

Patient	Diagnostic Diameter	Max. von Mises Stress	Max. Equivalent Strain	Displacement
**T1-P2**	48 mm	0.35 MPa	50%	13 mm
**T1-P5**	51 mm	0.37 MPa	50%	9 mm
**T1-P6-8**	58 mm	0.6 MPa	80%	24 mm

**Table 4:** Characteristics of examined aortas: T1-P9, T1-P10, and T1-P11.

Patient	Diagnostic Diameter	Max. von Mises Stress	Max. Equivalent Strain	Displacement
**T1-P9**	68 mm	0.35 MPa	50%	18 mm
**T1-P10**	48 mm	0.55 MPa	70%	35 mm
**T1-P11**	50 mm	0.25 MPa	40%	12 mm

**Table 5:** Characteristics of examined aortas: T1-P14, T1-P16, and T2-P2.

Patient	Diagnostic Diameter	Max. von Mises Stress	Max. Equivalent Strain	Displacement
**T1-P14**	50 mm	0.45 MPa	60%	21 mm
**T1-P16**	55 mm	0.4 MPa	50%	9 mm
**T2-P2**	49 mm	0.7 MPa	70%	14 mm

**Table 6:** Characteristics of examined aortas: T2-P3, T2-P13, and T2-P14.

Patient	Diagnostic Diameter	Max. von Mises Stress	Max. Equivalent Strain	Displacement
**T2-P3**	61 mm	0.3 MPa	35%	11 mm
**T2-P13**	51 mm	0.3 MPa	35%	14 mm
**T2-P14**	50 mm	0.4 MPa	50%	18 mm

**Table 7:** Risk Probability of all examined aortas.

Patient	Systolic Diameter	Pressure–Strain Modulus	Risk Probability
**T1-P2**	66	101.54	82.20%
**T1-P5**	63	140.00	90.74%
**T1-P6**	82	68.33	68.70%
**T1-P9**	86	95.56	80.30%
**T1-P10**	83	47.43	55.35%
**T1-P11**	55	91.67	78.95%
**T1-P14**	71	67.62	68.32%
**T1-P16**	64	142.22	91.09%
**T2-P2**	98	140.00	90.74%
**T2-P3**	72	130.91	89.20%
**T2-P13**	65	92.86	79.37%
**T2-P14**	68	75.56	72.32%

## Data Availability

Data are available from the corresponding author after reasonable request.
